# Supplementary material for: Contrastive learning explains the emergence and function of visual category-selective regions
Source: Sci Adv. 2024 Sep 25;10(39):eadl1776. doi: 10.1126/sciadv.adl1776 (PMC11423896; doi:10.1126/sciadv.adl1776)
Supplement: Supplementary file 1 — Figs. S1 to S13 [file sciadv.adl1776_sm.pdf]

Science Advances
AAAS

Supplementary Materials for

Contrastive learning explains the emergence and function of visual category-selective regions

Jacob S. Prince *et al.*

Corresponding author: Jacob S. Prince, jacob.samuel.prince@gmail.com

Sci. Adv. **10**, eadl1776 (2024)
DOI: 10.1126/sciadv.adl1776

This PDF file includes:

Figs. S1 to S13

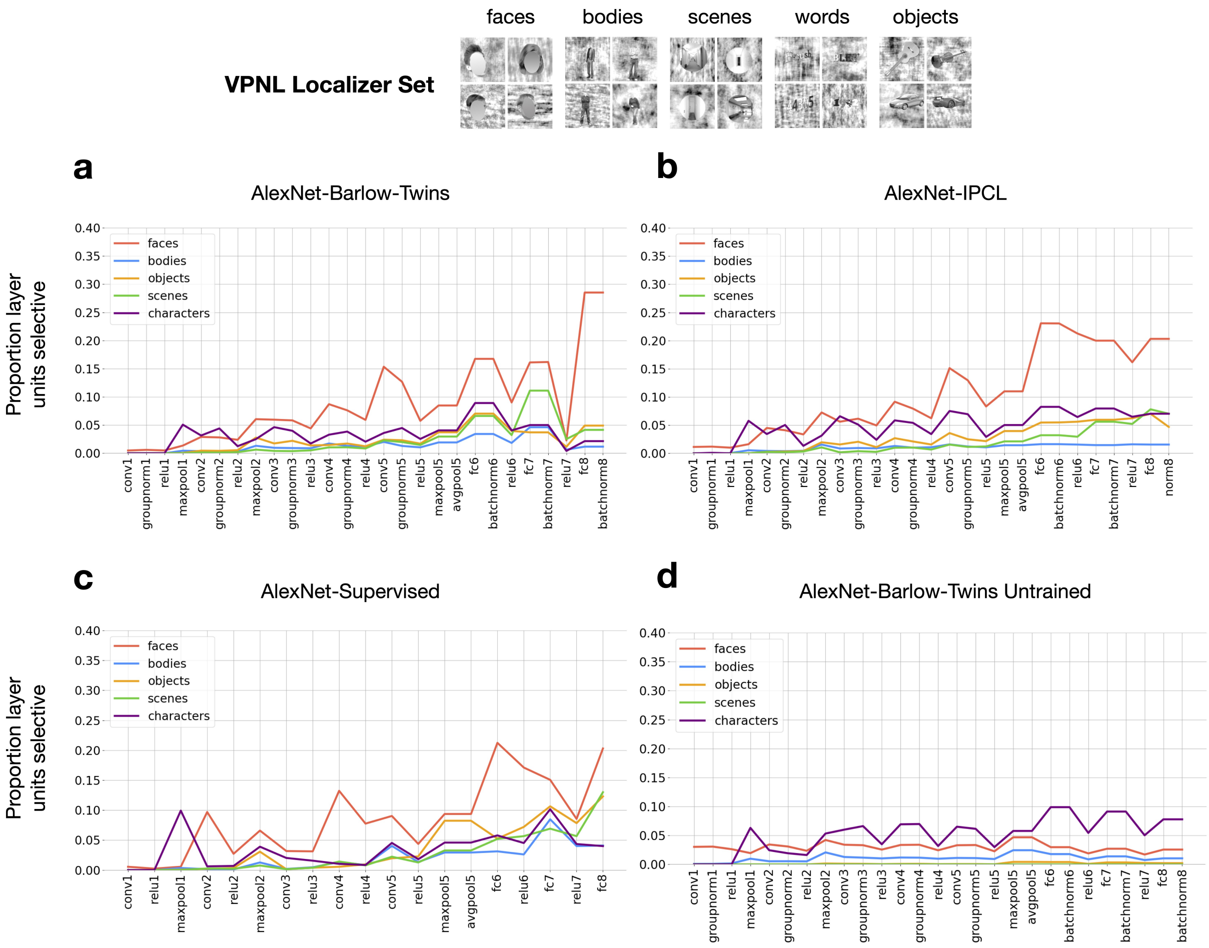

Supplementary Figure 1: Layer summary of localizer outcomes across DNNs. *Layerwise proportions of units selective for the 5 localizer domains are plotted for the trained AlexNet Barlow Twins model (top left), the AlexNet IPCL model (top right), a category-supervised AlexNet ImageNet model (TorchVision pretrained implementation; bottom left), and an untrained initialization of the Barlow Twins model (bottom right). Images containing identifiable human faces are masked.*

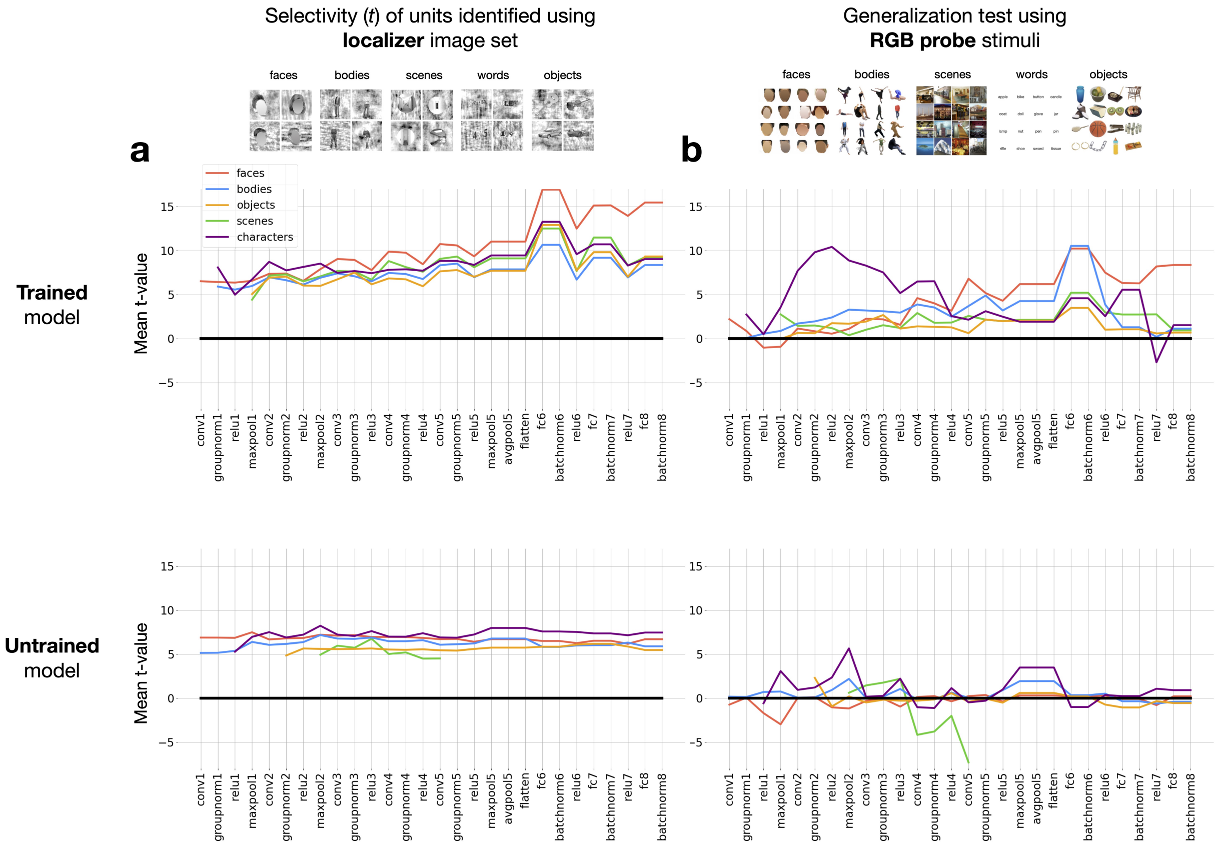

Supplementary Figure 2: Selectivity profiles do not generalize in an untrained DNN. *(A) Strength of units' selectivities for their preferred domains from the initial localizer run (vpnl-fLoc stimuli). Selectivities are summarized across model layers for the trained AlexNet Barlow Twins model (top) and the untrained model (bottom). The y-axis shows the mean t-statistic within each group of selective units (averaged both over units and over all statistical contrasts involving the target domain, e.g. faces vs. scenes, faces vs. bodies, etc.) (B) Generalization of selectivity to the independent RGB probe set. The t-values are reported from the same groups of units (defined using the vpnl-floc set), with the contrast t-values now computed over activations from the RGB image set. Images containing identifiable human faces are masked.*

a

Comparison between DNN unit selectivity criteria

	Faces			Bodies			Scenes			Words		
	2:1	t-test	Overlap	2:1	t-test	Overlap	2:1	t-test	Overlap	2:1	t-test	Overlap
relu7	5.91%	2.27%	31.89%	9.11%	0.63%	6.68%	9.96%	2.51%	18.84%	3.91%	0.42%	5.36%
**relu6**	**9.72%**	**9.01%**	**55.26%**	**8.57%**	**1.83%**	**15.45%**	**6.35%**	**3.22%**	**30.23%**	**4.10%**	**4.05%**	**34.68%**
relu5	3.86%	5.75%	30.07%	4.43%	1.05%	11.52%	2.25%	1.48%	19.60%	0.73%	2.54%	13.29%
relu4	4.36%	5.92%	41.21%	2.87%	1.21%	17.06%	0.99%	0.85%	27.70%	0.67%	2.05%	13.43%
relu3	3.02%	4.40%	43.04%	1.60%	0.89%	23.23%	0.90%	0.51%	21.53%	0.71%	1.73%	17.76%
relu2	3.15%	2.39%	40.86%	2.28%	0.30%	7.98%	0.86%	0.19%	11.04%	0.36%	1.25%	9.19%
relu1	0.30%	0.50%	18.73%	0.17%	0.01%	1.22%	0.05%	0.00%	0.00%	0.15%	0.01%	3.40%

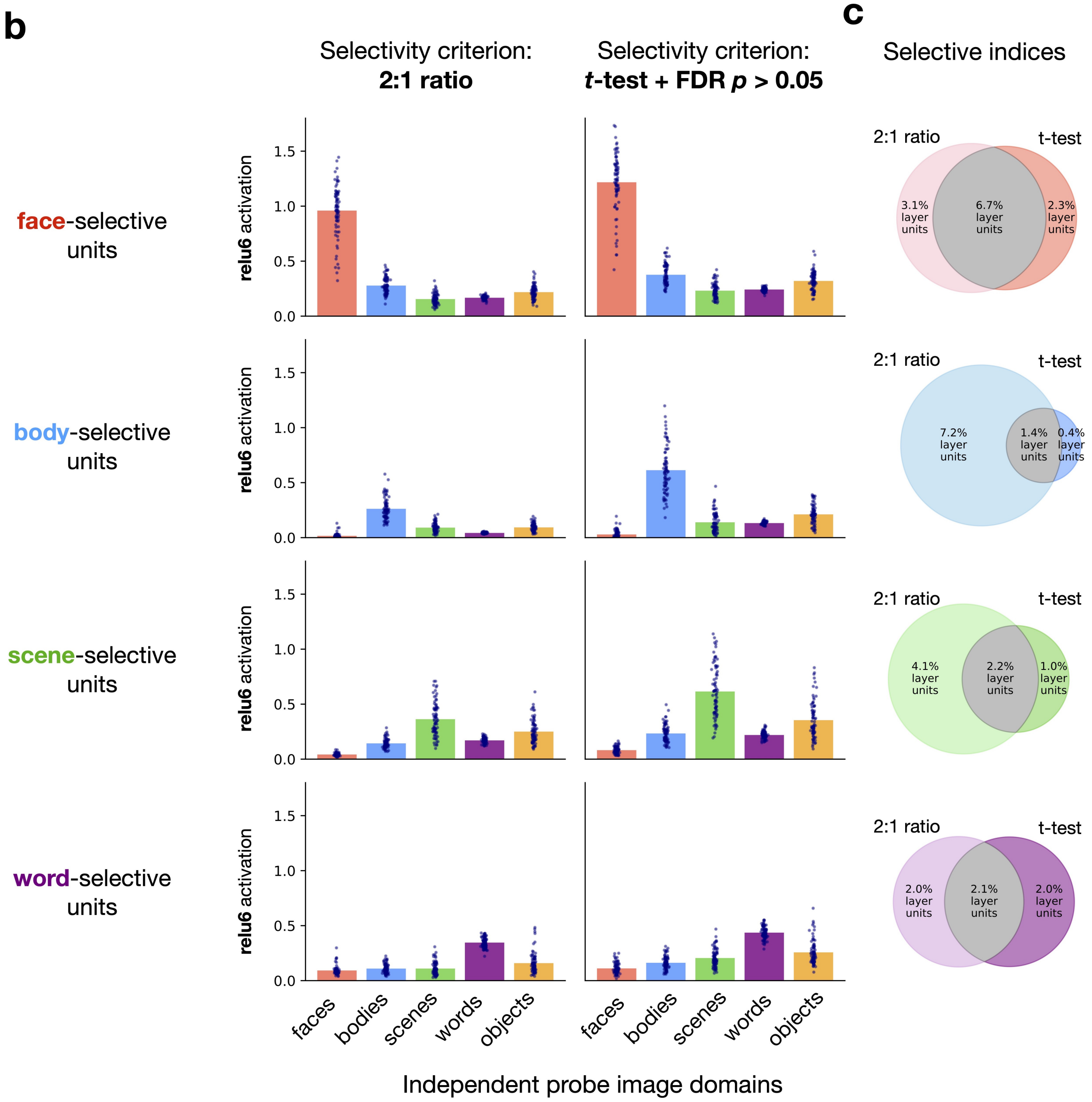

Supplementary Figure 3: Comparison of selectivity criteria. *(A) Outcomes of the 2:1 ratio and the t-test + FDR methods for defining selectivity. Proportions of selective units are shown for all relu layers of the AlexNet Barlow Twins model. 'Overlap' refers to the intersection over union of unit indices. (B) Comparing selective unit activations to RGB probe localizer images in layer relu6, between selectivity criteria. Bars reflect the mean activation within each group of units over the 80 images from each localizer domain. Image-wise means are plotted as dots. (C) Overlap of selective unit indices between the selectivity criteria, shown as venn diagrams. Units are expressed as proportions of the overall relu6 layer.*

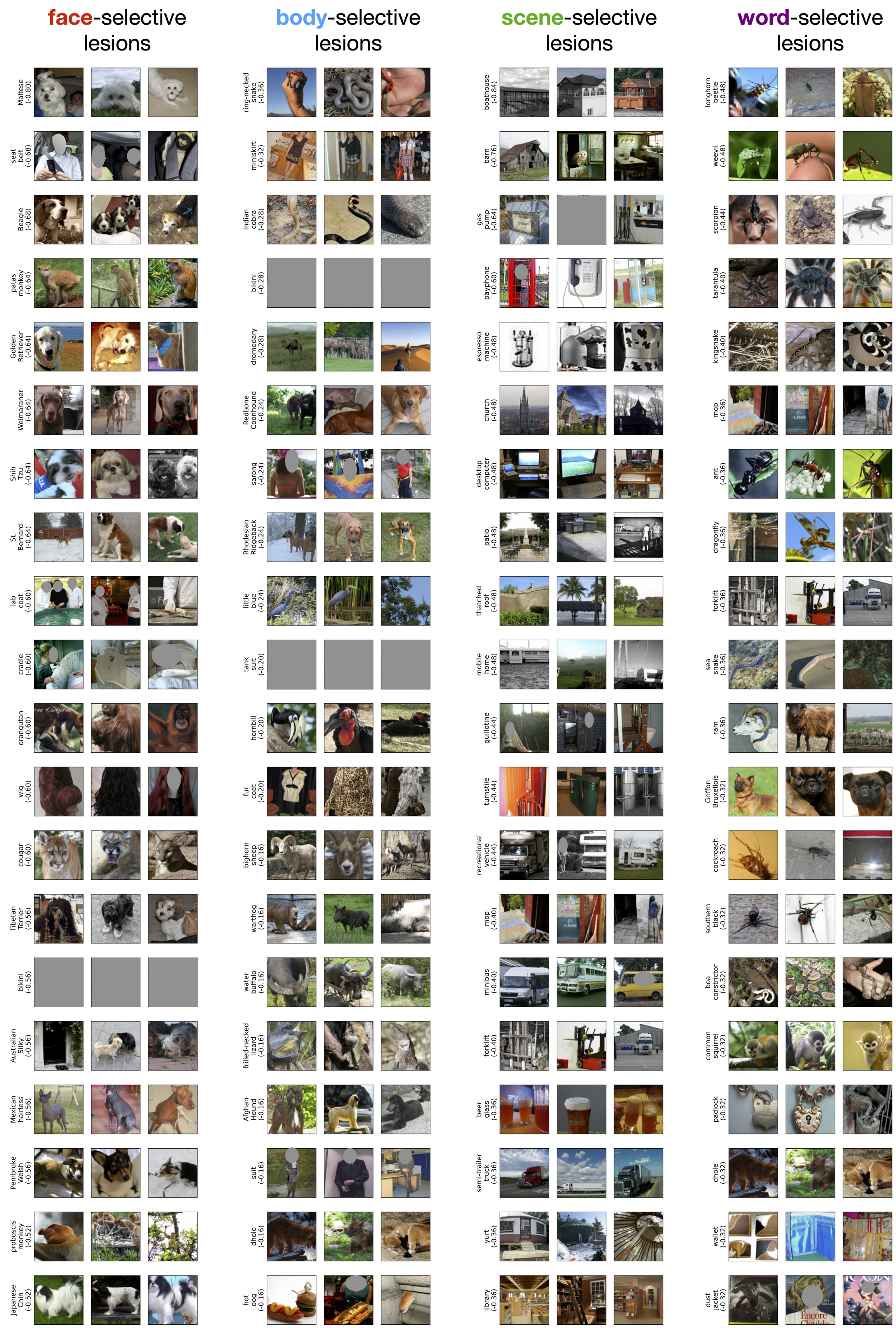

Supplementary Figure 4: Categories with recognition most impaired by each lesion. *The top 20 categories most impaired by each lesion type are identified using cross-validation (see Methods). Photo credit: images are samples from the public ImageNet validation set, and those containing human faces are masked. Some images are fully obscured due to their content.*

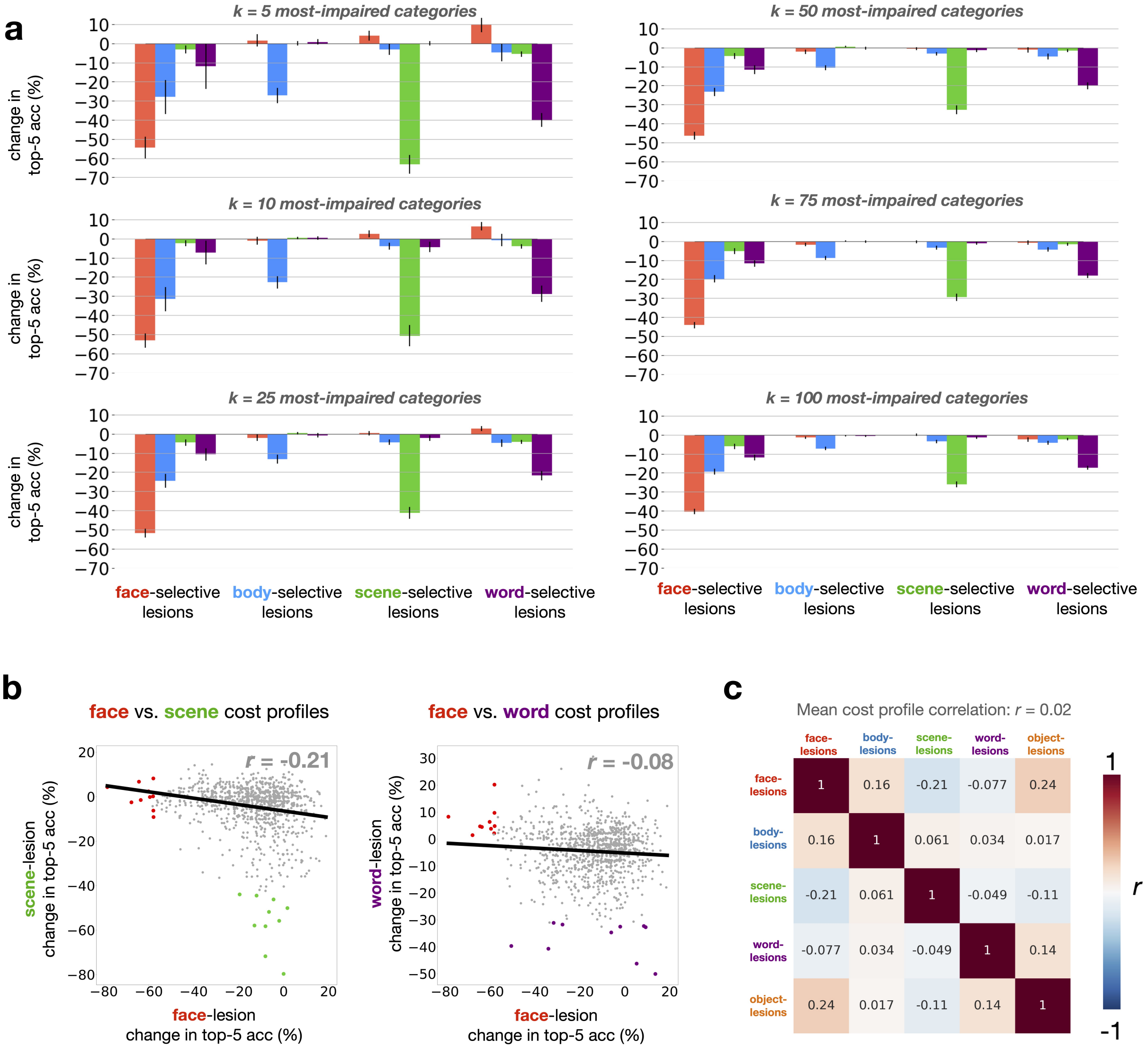

Supplementary Figure 5: Summary of dissociable lesioning deficits. *(A) Bar graphs show the mean (± SEM) change in % top-5 recognition accuracy over the top k categories most impacted by lesions to the selective units of each domain. Indices of the k categories are identified using half the ImageNet validation set, and accuracies for plotting are computed using the other half. (B) Relationship between 1000-dimensional profile of category-level lesioning deficits for face vs. scene unit lesions (left) and face vs. word unit lesions (right). Colored dots reflect the top 10 categories impacted by each domain's lesion. X and Y values are jittered (values drawn from normal distribution; mean 0, std 0.5%) to enhance visibility of the results. (C) Full pairwise similarity matrix (r) comparing the 1000-dim profiles of lesioning deficits that arise from lesions to each domain of selective units.*

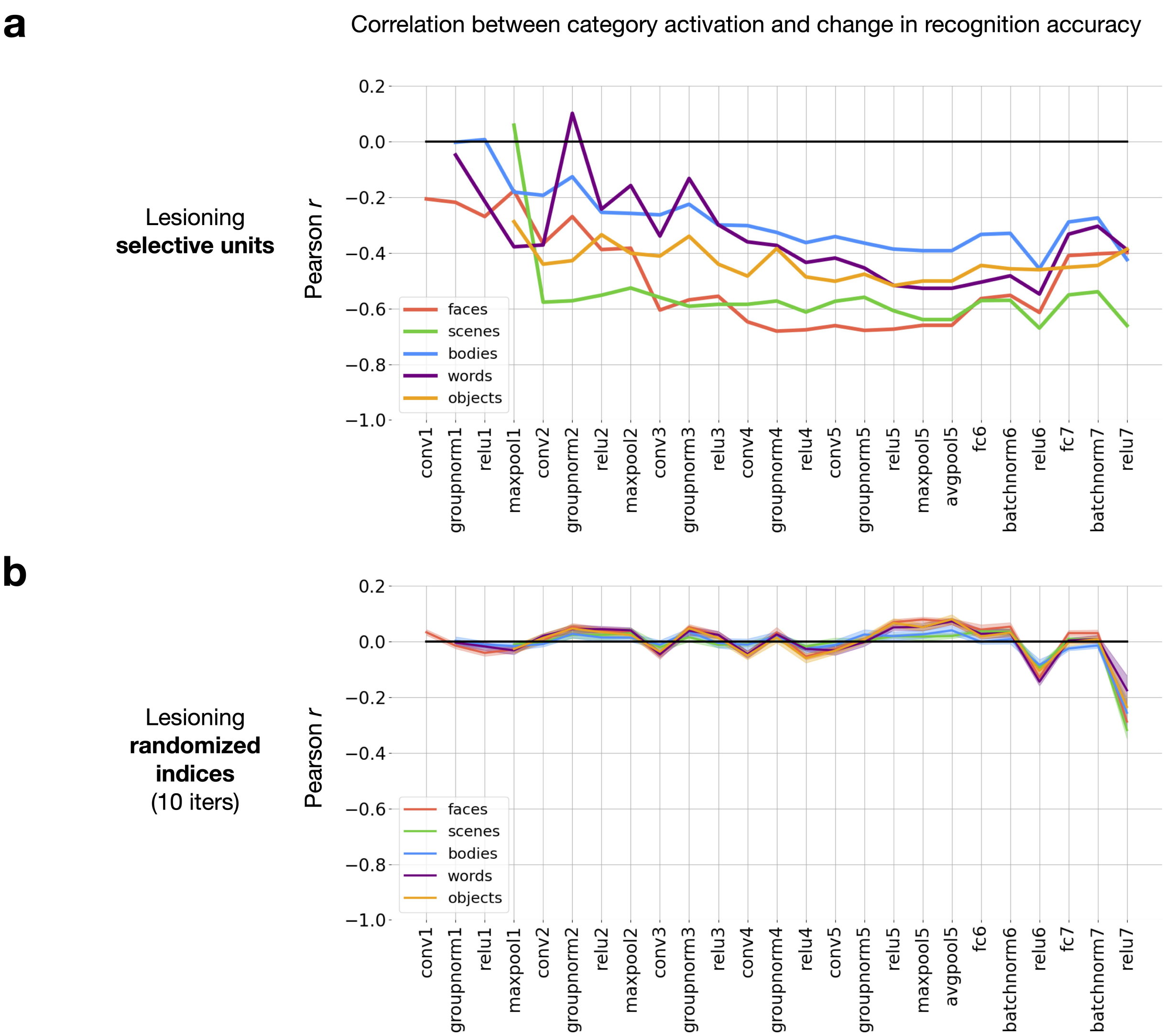

Supplementary Figure 6: Relationship between activation and recognition deficit for selective-unit and randomized lesions. *(A) Layer summary of the relationship between 1000-category profiles of mean activation magnitude (in the unlesioned model) and the changes in recognition accuracy observed after lesioning each domain's selective units. Data plotted are from the AlexNet Barlow Twins model, and lesions are applied to layers relu1-7. (B) The same analysis as (A), except that the indices of target units are randomized (within each layer) prior to lesioning. The randomized lesion experiment is repeated 10 times, with the mean ± SEM of resultant correlation values plotted.*

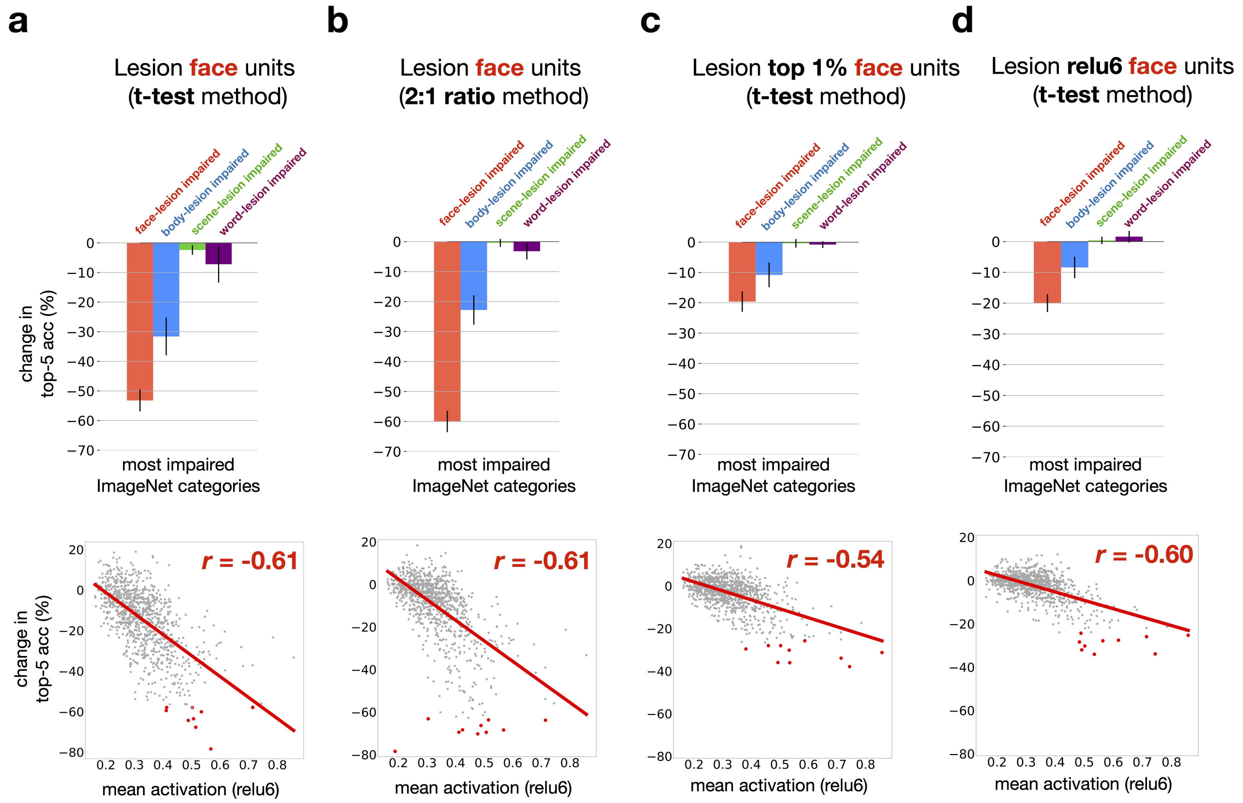

Supplementary Figure 7: Testing alternative lesioning schemes and results from 2:1 selectivity criterion. *(A) Top: changes in top-5 (%) accuracy are reported for the top 10 categories most impacted by face-selective unit lesions. Bottom: scatter plots show the relationship between mean activation pre-lesion and change in accuracy post-lesion for each of the 1000 ImageNet categories (dots). Same data as* ***Figure 2C****. Y values are jittered (values drawn from normal distribution; mean 0, std 0.5%) to enhance visibility of the results. (B) Results for face-selective units identified using the 2:1 ratio criterion, rather than the t-test + FDR localizer method. (C) Results for the t-test + FDR localizer method, with only the top 1% of most face-selective units in each relu layer lesioned. (D) Results for the t-test + FDR localizer method, with the full set of face-selective units from only layer relu6 lesioned.*

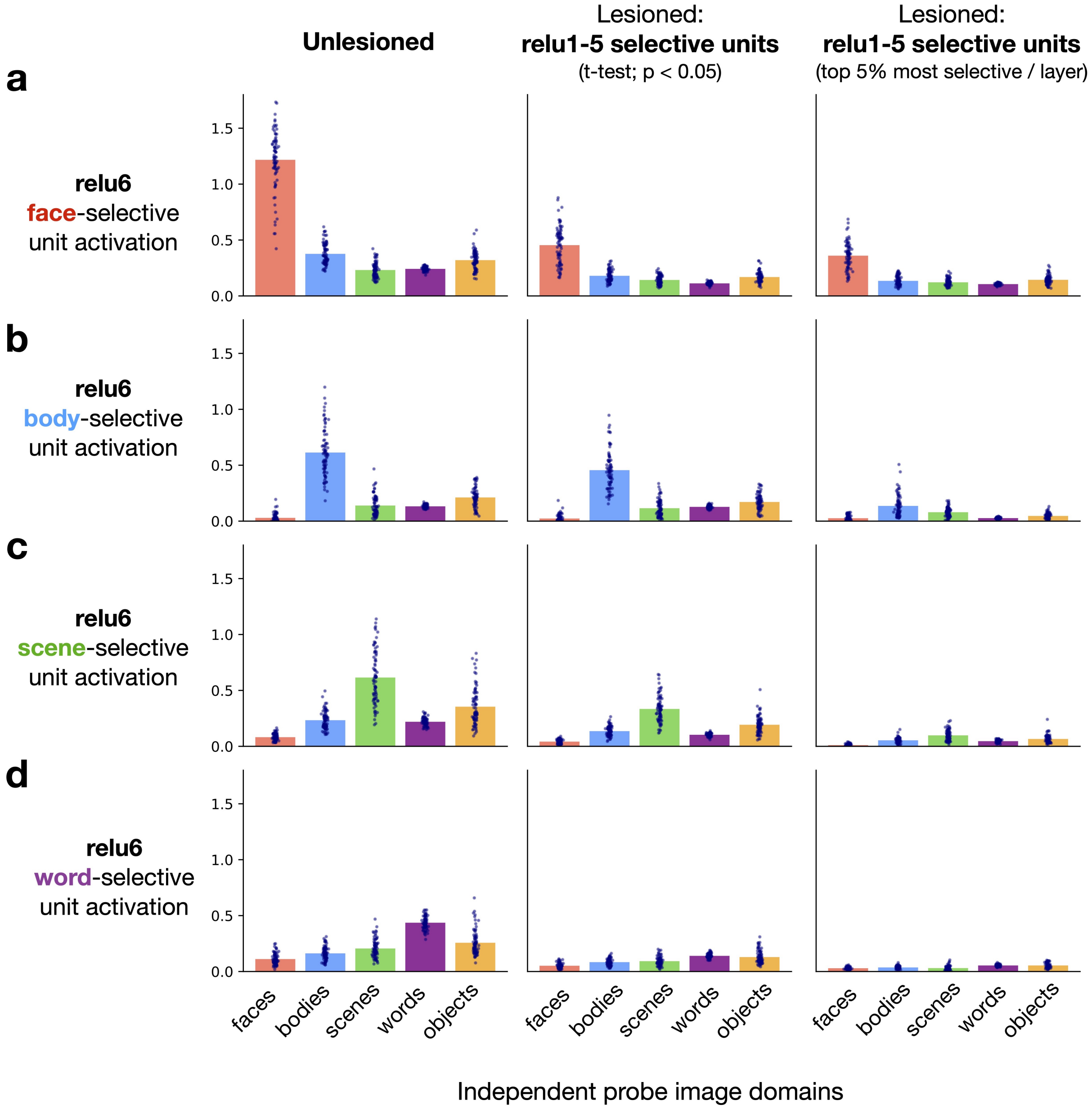

Supplementary Figure 8: Impact of lesioning on downstream selectivity. *(A) Mean activations are plotted in response to the RGB probe localizer images for face-selective units in layer relu6, identified using the t-test + FDR selectivity criterion. Left panel: activations with no lesions implemented (same data as* ***Supplementary Fig. 3B****). Middle panel: relu6 activations after lesions are applied to the face-selective units of relu layers 1-5, with layer relu6 unlesioned. Right panel: Same as middle panel, except with only the top 5% of selective units lesioned in each relu layer 1-5. (B-D) The same analyses, repeated for the body-, scene-, and word-selective units.*

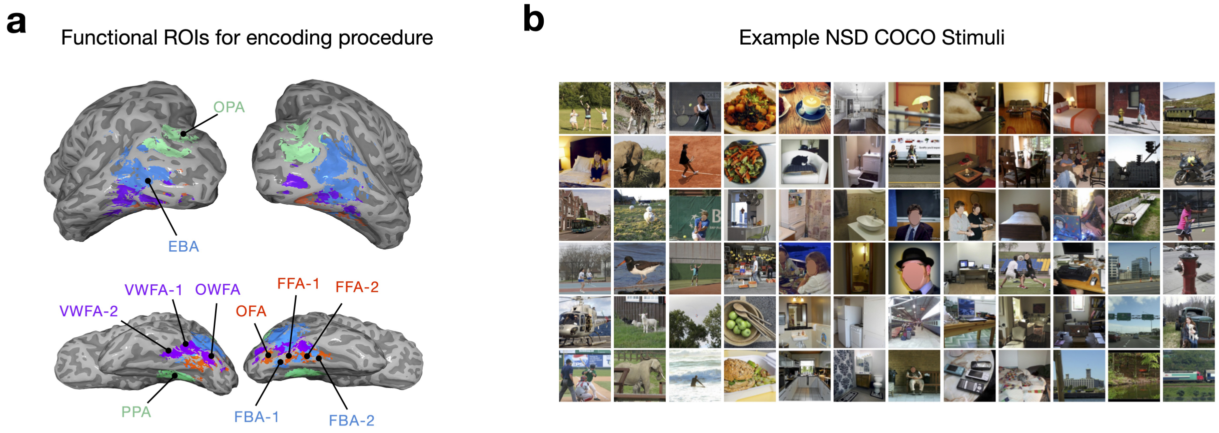

Supplementary Figure 9: Overview of functional ROIs and NSD stimuli. *(A) Data from the Natural Scenes Dataset (N = 8; representative subject plotted) used for the encoding procedure. 11 category selective ROIs are analyzed per subject, with preferences for faces (red), scenes (green), bodies (blue), and words (purple). (B) Examples from the test set of 515 stimuli from the NSD experiment. Photo credit: images in Panel B are samples from the public Microsoft COCO dataset, and those containing human faces are masked.*

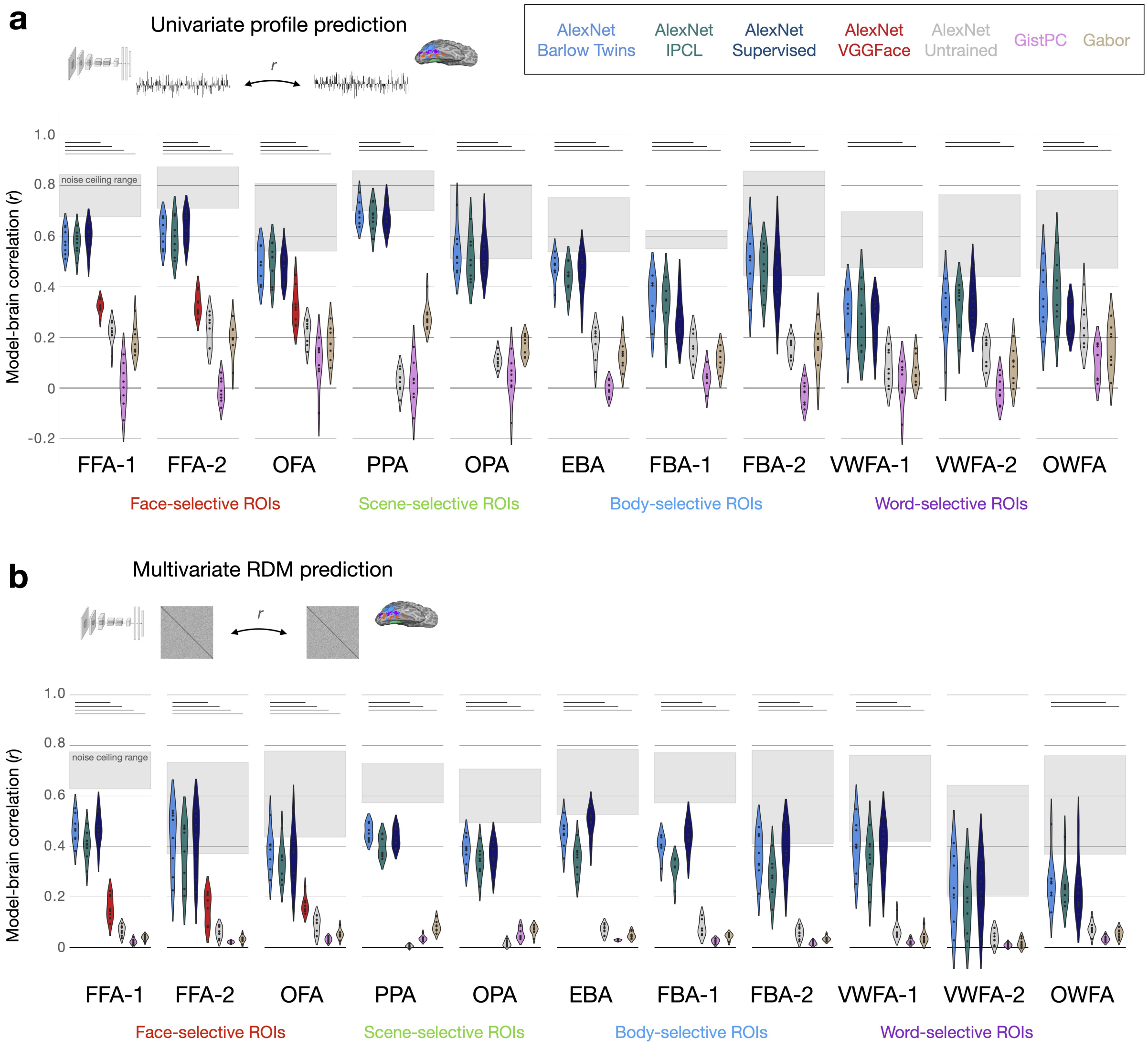

Supplementary Figure 10: Brain prediction summary across a larger set of selective regions. *Encoding results for predicting univariate response profiles (A) and multivariate RDMs (B) are summarized for the 8 NSD subjects, for each DNN and low-level image statistic model. Plotted values reflect best-layer correlations, as defined using cross-validation (see Methods) Shaded regions show the range of subject-specific noise ceilings computed over the data from each ROI. Significance is assessed using paired* t*-tests over subject-wise prediction levels between the AlexNet Barlow Twins model and each other candidate model. Horizontal bars reflect significant effects favoring the Barlow Twins model; Bonferroni-corrected* $p < 0.001$.

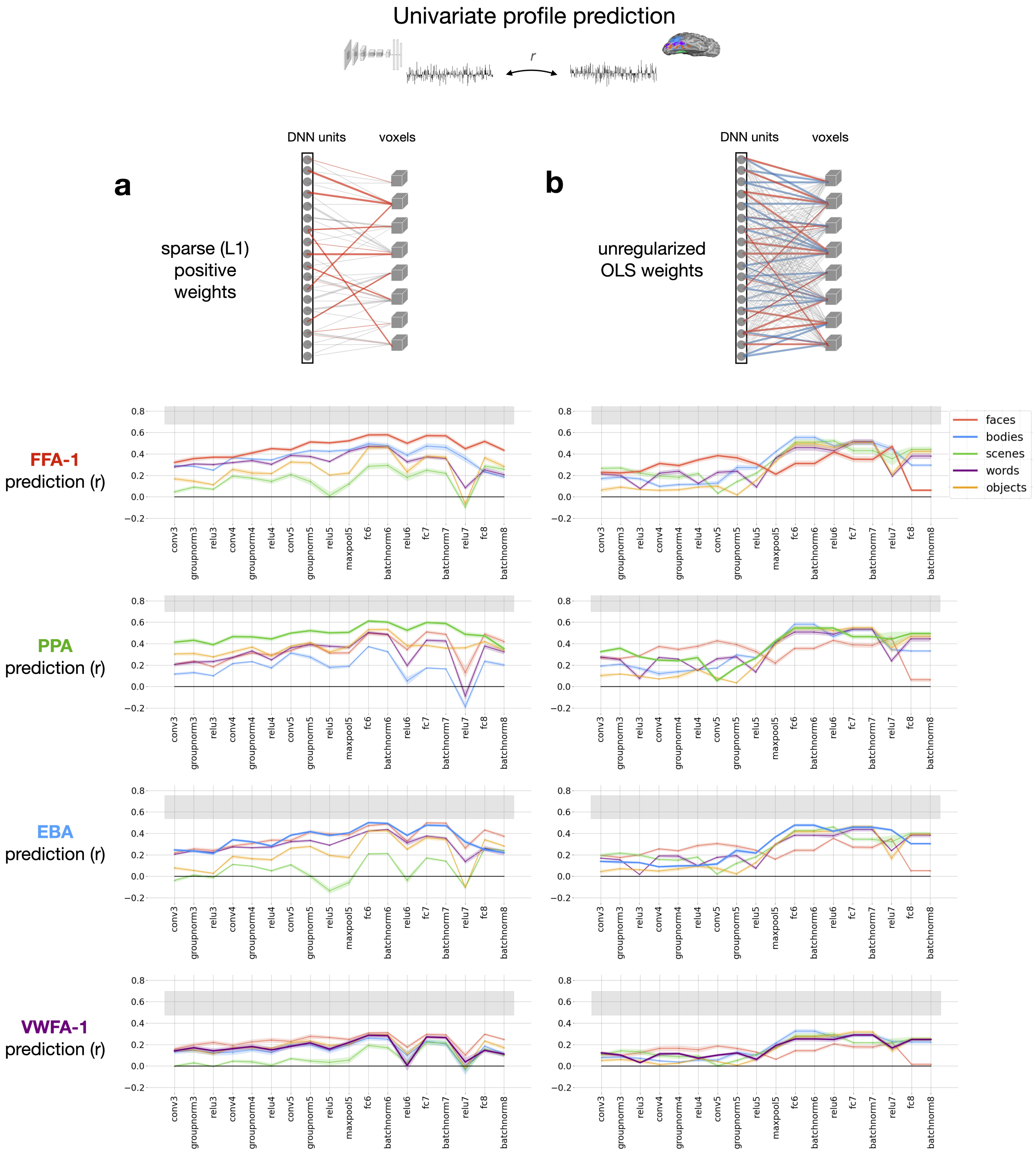

Supplementary Figure 11: Impact of sparse-positive readout (univariate profile prediction). *Layer summaries of encoding results are plotted for prediction of univariate response profiles in FFA-1, PPA, EBA, and VWFA-1 (N = 8 subjects, n = 515 test images). All data are from the AlexNet Barlow Twins model, with line colors representing the domain of selective units that are mapped onto the target ROI. Prediction results are compared between (A) the constrained encoding procedure involving sparse-positive weights only, and (B) an unregularized encoding procedure that uses ordinary least-squares (OLS) fitting, with no additional constraints. Shaded regions reflect the range of subject-specific noise ceilings computed over the univariate response profiles from each ROI.*

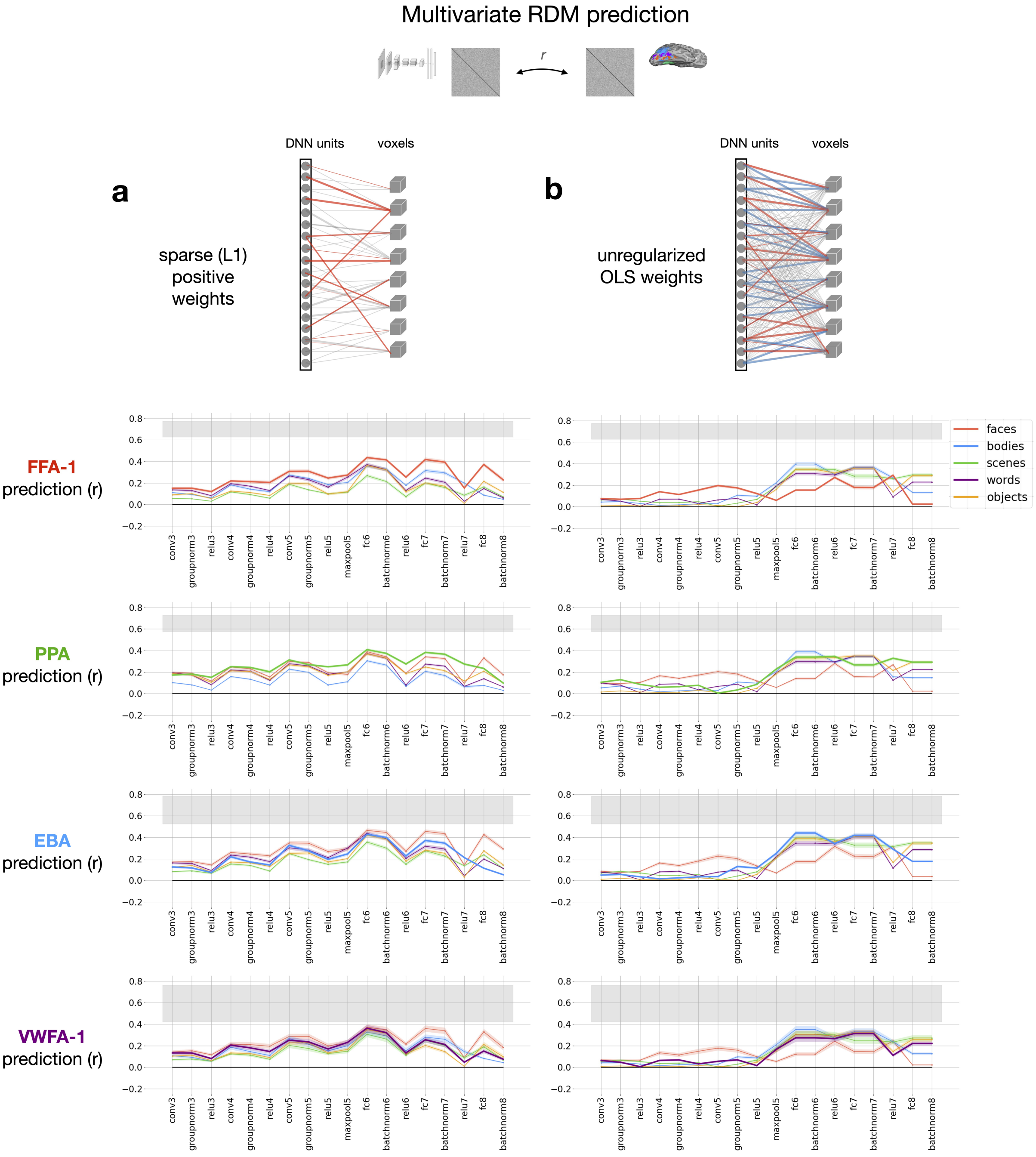

Supplementary Figure 12: Impact of sparse-positive readout (multivariate RDM prediction). *Layer summaries of encoding results are plotted for prediction of multivariate RDMs in FFA-1, PPA, EBA, and VWFA-1 (N = 8 subjects, n = 132,355 unique pairwise comparisons per RDM). All data are from the AlexNet Barlow Twins model, with line colors representing the domain of selective units that are mapped onto the target ROI. Prediction results are compared between (A) the constrained encoding procedure involving sparse-positive weights only, and (B) an unregularized encoding procedure that uses ordinary least-squares (OLS) fitting, with no additional constraints. Shaded regions reflect the range of subject-specific noise ceilings computed over the RDMs from each ROI.*

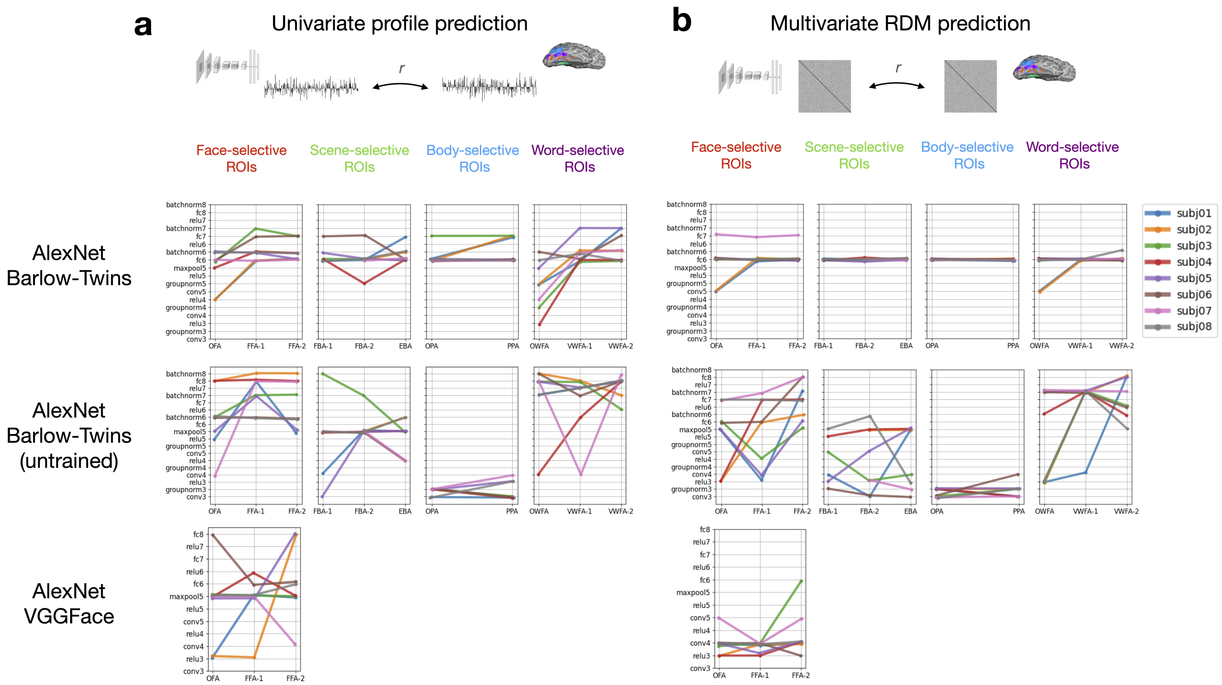

Supplementary Figure 13: Indices of the most brain-predictive DNN layers. *Indices of the most predictive DNN layers for univariate (A) and multivariate (B) encoding are identified using a validation set of 1000 subject-specific NSD stimuli. Lines reflect the outcomes for the 8 NSD subjects, across all 11 functional ROIs, for the three DNN models included in the encoding procedure.*
